# Supplementary material for: Finding haplotypic signatures in proteins
Source: Gigascience. 2023 Oct 31;12:giad093. doi: 10.1093/gigascience/giad093 (PMC10622322; doi:10.1093/gigascience/giad093)
Supplement: giad093_Response_to_Reviewer_Comments_Original_Submission [file giad093_response_to_reviewer_comments_original_submission.pdf]

Author's Response To Reviewer Comments

Please see supplementary files for our response to reviewers and tables.
Also a version showing our edits.
