# Supplementary material for: Finding haplotypic signatures in proteins
Source: Gigascience. 2023 Oct 31;12:giad093. doi: 10.1093/gigascience/giad093 (PMC10622322; doi:10.1093/gigascience/giad093)
Supplement: giad093_Response_to_Reviewer_Comments_Revision_1 [file giad093_response_to_reviewer_comments_revision_1.pdf]

Author's Response To Reviewer Comments

Dear editor,

We have edited the manuscript according to your suggestions. The only issue is that we could not get an RRID from SciCrunch, it seems that the system does not work and we did not get any answer from the support.

Many thanks for all the work that you put in our manuscript.

On behalf of the authors,

Marc
