# Supplementary material for: Finding haplotypic signatures in proteins
Source: Gigascience. 2023 Oct 31;12:giad093. doi: 10.1093/gigascience/giad093 (PMC10622322; doi:10.1093/gigascience/giad093)
Supplement: giad093_Reviewer_1_Report_Original_Submission — Teck Yew Low -- 5/5/2023 Reviewed [file giad093_reviewer_1_report_original_submission.pdf]

Reviewer Report

Title: Finding Haplotypic Signatures in Proteins

Version: Original Submission **Date:** 5/5/2023

Reviewer name: Teck Yew Low

Reviewer Comments to Author:

This is a very interesting manuscript that describe the incorporation of haplotypes into protein fasta which would enable more precise and specific database searching for bottom-up proteomics MS data. It also discusses certain pitfalls that come along with intended improvement in data resolution i.e. the mis-assignment of amino acid substitutions with other amino acids or PTMs that are very close in mass. The manuscript is well-written and the reasonings are clearly explained. Besides, it does provide new insights into providing more accurate search at a proteogenome level.
My comment as as follows:
(i) In the introduction, the authors mentioned that when haplotypes are present in protein coding regions, they can alter protein sequences. To be more precise, only the non-synonymous ones can alter amino acid sequences.
(ii) Only tryptic peptides of 8-40 amino acids were in silico translated, why not start with 6 or 7 amino acids which are commonly adopted by the community?
(iii) The peptides were categorized into any categories and each sub-category has its number. This is quite confusing. May be the author can generate a table to host all these categories and its associating numbers
(iv) While I do acknowledge that challenges exist to distinguish peptides (with aa substitution and PTMs) that are very close in mass, is it possible to run search with different search engines for example MaxQuant or MSFragger etc as it is well-known that there are some discrepancy in PSM matching among different search engines.
(v) Further, these search engines provide "second peptide" feature to resolve chimeric spectra and "open search" feature to allow searching unknown PTMs.
(vi) Also how prevalent are such mass ambiguity? Failure to assign exact amino acid sequences may only constitute a minor % of spectra?
(vii) Have you come across peptides that can be clearly assigned, that have come from paternal allele and maternal allele within a single sample?

Methods

Are the methods appropriate to the aims of the study, are they well described, and are necessary controls included? Choose an item.

Conclusions

Are the conclusions adequately supported by the data shown? Choose an item.

Reporting Standards

Does the manuscript adhere to the journal's guidelines on minimum standards of reporting? Choose an item.

Choose an item.

Statistics

Are you able to assess all statistics in the manuscript, including the appropriateness of statistical tests used? Choose an item.

Quality of Written English

Please indicate the quality of language in the manuscript: Choose an item.

Declaration of Competing Interests

Please complete a declaration of competing interests, considering the following questions:

- Have you in the past five years received reimbursements, fees, funding, or salary from an organisation that may in any way gain or lose financially from the publication of this manuscript, either now or in the future?
- Do you hold any stocks or shares in an organisation that may in any way gain or lose financially from the publication of this manuscript, either now or in the future?
- Do you hold or are you currently applying for any patents relating to the content of the manuscript?
- Have you received reimbursements, fees, funding, or salary from an organization that holds or has applied for patents relating to the content of the manuscript?
- Do you have any other financial competing interests?
- Do you have any non-financial competing interests in relation to this paper?

If you can answer no to all of the above, write 'I declare that I have no competing interests' below. If your reply is yes to any, please give details below.

I declare that I have no competing interests

I agree to the open peer review policy of the journal. I understand that my name will be included on my report to the authors and, if the manuscript is accepted for publication, my named report including any attachments I upload will be posted on the website along with the authors' responses. I agree for my report to be made available under an Open Access Creative Commons CC-BY license (http://creativecommons.org/licenses/by/4.0/). I understand that any comments which I do not wish to be included in my named report can be included as confidential comments to the editors, which will not be published.

Choose an item.

To further support our reviewers, we have joined with Publons, where you can gain additional credit to further highlight your hard work (see: https://publons.com/journal/530/gigascience). On publication of this paper, your review will be automatically added to Publons, you can then choose whether or not to claim your Publons credit. I understand this statement.

Yes Choose an item.
