# Supplementary material for: Finding haplotypic signatures in proteins
Source: Gigascience. 2023 Oct 31;12:giad093. doi: 10.1093/gigascience/giad093 (PMC10622322; doi:10.1093/gigascience/giad093)
Supplement: giad093_Reviewer_2_Report_Original_Submission — Subina Mehta, M.S -- 6/23/2023 Reviewed [file giad093_reviewer_2_report_original_submission.pdf]

Reviewer Report

Title: Finding Haplotypic Signatures in Proteins

Version: Original Submission **Date:** 6/23/2023

Reviewer name: Subina Mehta, M.S

Reviewer Comments to Author:

This manuscript by VaÅ¡Ã-Ä□ek et.al. offers valuable perspectives on the significance of examining haplotypes, as they play a crucial role in identifying genes and its locations responsible for diseases. There are not a lot of studies on haplotypes using mass spectrometry in comparison to genomic studies. The various methods for classifying protein haplotypes according to peptide variants have been outlined in the research paper. The validation of haplotype peptide variants in the study using PepQuery is an intriguing observation. Cancer proteogenomics research will greatly benefit from the findings of this study.

Few minor edits are recommended.

1) Figure 2: "height of the bars represents the distribution" rather than "height of the bars represent the distribution"
2) Page 8, Paragraph 2- "As highlighted by Spooner"- in line "These haplotypes encode a tryptic peptide containing two amino acid
substitutions when compared to the canonical sequence in Ensembl" - Please mention the peptide and bold the variant amino acids.
3) Figure 4,5 and 7 could be enlarged a bit to see the PSM.
4) Page 12- "PepQuery found that a substantial share of the
matches were low scoring or could also match another peptide" - what would categorize as a low score? p value or PepQuery score?
5) Haplosaurus tool is mentioned but no repository or link is provided.
6) In Materials and Methods- FoO abbreviation is mentioned but not full form. Also missing from the abbreviations section.
7) Public data reanalysis- It would be helpful if a supplementary table is added providing the search parameters for the data analysis/database searching .
8) Public data reanalysis- As SearchGUI and Peptide Shaker are used for the analysis, it is essential to mention the version numbers of these tools.
9) PepQuery Analysis- "Ammonia- loss of C, Glu->pyro-Glu of E, Gln->pyro-Glu of Q, Acetylation of peptide N-term; " if these modifications are used in PepQuery, then were these used during Database searching also with SearchGUI Peptide Shaker?
10) PepQuery analysis- What were the filtering criteria used? which PepQuery scoring algorithm was used? it is not clear whether for filtering aa substitution modifications were considered or not or was it filtered only by unmodified search.

Methods

Are the methods appropriate to the aims of the study, are they well described, and are necessary controls included? Choose an item.

Conclusions

Are the conclusions adequately supported by the data shown? Choose an item.

Reporting Standards

Does the manuscript adhere to the journal's guidelines on minimum standards of reporting? Choose an item.

Choose an item.

Statistics

Are you able to assess all statistics in the manuscript, including the appropriateness of statistical tests used? Choose an item.

Quality of Written English

Please indicate the quality of language in the manuscript: Choose an item.

Declaration of Competing Interests

Please complete a declaration of competing interests, considering the following questions:

- Have you in the past five years received reimbursements, fees, funding, or salary from an organisation that may in any way gain or lose financially from the publication of this manuscript, either now or in the future?
- Do you hold any stocks or shares in an organisation that may in any way gain or lose financially from the publication of this manuscript, either now or in the future?
- Do you hold or are you currently applying for any patents relating to the content of the manuscript?
- Have you received reimbursements, fees, funding, or salary from an organization that holds or has applied for patents relating to the content of the manuscript?
- Do you have any other financial competing interests?
- Do you have any non-financial competing interests in relation to this paper?

If you can answer no to all of the above, write 'I declare that I have no competing interests' below. If your reply is yes to any, please give details below.

I declare that I have no competing interests.

I agree to the open peer review policy of the journal. I understand that my name will be included on my report to the authors and, if the manuscript is accepted for publication, my named report including any attachments I upload will be posted on the website along with the authors' responses. I agree for my report to be made available under an Open Access Creative Commons CC-BY license (http://creativecommons.org/licenses/by/4.0/). I understand that any comments which I do not wish to be included in my named report can be included as confidential comments to the editors, which will not be published.

Choose an item.

To further support our reviewers, we have joined with Publons, where you can gain additional credit to further highlight your hard work (see: https://publons.com/journal/530/gigascience). On publication of this paper, your review will be automatically added to Publons, you can then choose whether or not to claim your Publons credit. I understand this statement.

Yes Choose an item.
